# Boron Neutron Capture Therapy in Recurrent High-Grade Gliomas: Safety, Efficacy, and Pharmacokinetics From a Multicenter, Dose-Escalation Phase 1 Trial

**DOI:** 10.1016/j.adro.2025.101947

**Published:** 2025-10-30

**Authors:** Woohyoung Kim, Jae-Sung Park, Jin-Ho Song, Heon Yoo, Kawngwoo Park, Hyun Ju Kim, Dong-Won Shin, Sung Uk Lee, Stephen Ahn, Seunggyun Ha, JunGyu Yi, Kwan Cho, Hyo Jung Seo, Hyung-Seok Lim, Gi-Taek Yee

**Affiliations:** a Department of Clinical Development, DAWONMEDAX CO, Ltd, Incheon, Republic of Korea; b Department of Neurosurgery, Seoul St. Mary's Hospital, College of Medicine, The Catholic University of Korea, Seoul, Republic of Korea; c Department of Radiation Oncology, Seoul St. Mary's Hospital, College of Medicine, The Catholic University of Korea, Seoul, Republic of Korea; d Department or Neuro-Oncology Clinic, National Cancer Center, Goyang, Republic of Korea; e Department of Neurosurgery, Gachon University Gil Medical Center, Incheon, Republic of Korea; f Department of Radiation Oncology, Gachon University Gil Medical Center, Gachon University College of Medicine, Incheon, Republic of Korea; g Department of Neurosurgery, Gachon University Gil Medical Center, Gachon University College of Medicine, Incheon, Republic of Korea; h Center for Proton Therapy, National Cancer Center, Goyang, Republic of Korea; i Division of Nuclear Medicine, Department of Radiology, Seoul St. Mary's Hospital, College of Medicine, The Catholic University of Korea, Seoul, Republic of Korea; j Medical Device Division, DAWONMEDAX CO, Ltd, Incheon, Republic of Korea; k Department of Nuclear Engineering, Hanyang University, Seoul, Republic of Korea; l DAWONMEDAX CO, Ltd, Incheon, Republic of Korea; m Department of Nuclear Medicine, BNCT Center, Incheon, Republic of Korea; n Asan Medical Center, Department of Clinical Pharmacology and Therapeutics, University of Ulsan College of Medicine, Seoul, Republic of Korea; o Asan Medical Center, Department of Medical Science, Asan Medical Institute of Convergence Science and Technology, University of Ulsan College of Medicine, Seoul, Republic of Korea

## Abstract

**Purpose:**

Boron neutron capture therapy (BNCT) is an advanced radiation therapy delivering highly selective tumor cell destruction via localized fission reactions. This phase 1 study evaluated the safety and efficacy of BNCT using [B-10]L-4-boronophenylalanine (BPA) in patients with recurrent high-grade gliomas, mostly glioblastomas.

**Methods and Materials:**

A 3 + 3 dose-escalation design was used to determine the maximum tolerated dose of BNCT across 3 planned cohorts (D_max_: 9, 11, and 13 Gy-Eq), with 3 additional subjects enrolled at any dose level where dose-limiting toxicity (DLT) occurred. DLT was defined as BNCT-related grade 3+ toxicities within 90 days posttreatment, with protocol-defined exclusions. Patients underwent a single session: intravenous BPA administration (500 mg/kg/3 h) and neutron irradiation 1 hour later. The primary endpoint was the recommended phase 2 radiation dose, based on DLT incidence. Secondary endpoint included safety, efficacy, and pharmacokinetics: treatment-emergent adverse events (TEAEs), progression-free survival, objective response rate, and overall survival (OS).

**Results:**

Six patients were treated between December 2022 and January 2024: 3 in the 9 Gy-Eq and 3 in the 11 Gy-Eq cohort. No DLTs or grade ≥4 TEAEs occurred. Two patients experienced grade 3 TEAEs (brain edema, seizure). Common adverse events were alopecia, aphasia, brain edema, and seizures. One serious adverse event (grade 3 seizure) was reported. Median follow-up was 9.03 months. Median progression-free survival was 1.87 months by response assessment in neuro-oncology; not reached by modified response assessment in neuro-oncology. Objective response was not observed. All patients survived to study completion; median OS not reached, maximum OS was 16.56 months. BPA pharmacokinetics were within expected ranges. The safety monitoring committee selected 11 Gy-Eq as the recommended phase 2 radiation dose, balancing tumoricidal effects with risks of necrosis and bevacizumab requirements.

**Conclusions:**

This study demonstrates the acceptable safety profile of BNCT and suggests potential survival benefits in recurrent high-grade glioma. However, given the limited sample size and follow-up period, extended observation is required to validate long-term efficacy and safety.

## Introduction

Boron neutron capture therapy (BNCT) has emerged as a promising therapeutic modality, leveraging tumor cell-selective radiation therapy through the capture reaction of boron-10 and thermal neutrons. The unique mechanism of BNCT enables precise targeting of tumor cells while sparing surrounding normal tissue, offering a potential advantage over conventional radiation therapy. Previous clinical studies have illustrated the feasibility and potential efficacy of BNCT in high-grade gliomas, underscoring its role as a viable option for patients with limited treatment alternatives.^1^, ^2^, ^3^, ^4^, ^5^

Glioblastoma (GBM), which constitutes a majority of high-grade gliomas, is a highly aggressive and treatment-resistant primary brain tumor. It is characterized by rapid progression and a generally poor prognosis. Despite advancements in standard treatment modalities, including gross total resection, radiation therapy, and chemotherapy with temozolomide, the median overall survival (OS) of the newly diagnosed patients with GBM remains approximately 12 to 15 months,^6^ with most patients experiencing recurrence within the first year. This highlights an urgent need for innovative therapeutic approaches that can improve outcomes for these patients.

This study represents a phase 1 clinical trial aimed to evaluate the safety and preliminary efficacy of BNCT in patients with recurrent high-grade gliomas, specifically GBM. The primary objective of this trial was to determine the maximum tolerable dose (MTD) and confirm the recommended phase 2 radiation dose (RP2D). Secondary objectives were to assess safety, efficacy, and pharmacokinetic (PK) profiles of BNCT.

## Methods and Materials

### Study design

This study was designed as a multicenter, open-label, radiation dose-escalation phase 1 trial with the objective of evaluating the safety and preliminary efficacy of BNCT in patients who received a diagnosis of recurrent high-grade gliomas. The study employed a 3 + 3 dose-escalation design with 3 planned cohorts (targeting maximum brain dose [D_max_] of 9, 11, and 13 Gy-Eq) to determine the MTD of BNCT. An overview of the protocol is shown in Fig. E1. Safety monitoring committee (SMC) confirmed the occurrence of dose-limiting toxicities (DLTs) based on BNCT-related adverse events (AEs) which occurred within 90 days post-BNCT and decided whether to escalate the next dose level or confirm the MTD. DLT was defined as, according to National Cancer Institute Common Terminology Criteria for Adverse Events version 5.0, a grade 3 or higher hematologic or nonhematologic toxicity that was related to BNCT and occurred within 90 days after treatment. The next dose level was proceeded only if fewer than 2 DLTs were observed in the cohort.

This study was conducted in accordance with the guidance of Good Clinical Practice as well as the Declaration of Helsinki. The study was approved by the institutional review board of each site and registered in ClinicalTrials.gov. (NCT05737212).

### Patient eligibility

Patients aged between 19 and 80 years with histologically or radiologically confirmed recurrent high-grade gliomas were eligible for inclusion in this study. The inclusion criteria were as follows: a Karnofsky performance status score of ≥60, prior standard radiation therapy (54-66 Gy/25-35 fractions) or less, and appropriate renal, hepatic, and hematologic function. Patients with uncontrolled systemic illnesses, persistent cerebral edema despite corticosteroid administration, significant laboratory abnormalities, or prior exposure to BNCT were excluded from this study.

### Treatment procedure

Patients received a single session of BNCT as part of this trial. The investigational drug, labeled as DMX-101, ^10^B-boronophenylalanine (BPA) (DAWONMEDAX CO. LTD., South Korea), was administered intravenously at a dose of 500 mg/kg over 3 hours. Neutron irradiation using DM-BNCT (DAWONMEDAX CO. LTD, South Korea), a medical linear accelerator, was initiated 1 hour after the completion of BPA infusion, a timing determined based on PK characteristics indicating that peak boron concentration within tumor tissues occurs approximately 1 hour postinfusion^7^ so that maximum therapeutic efficacy while minimizing off-target radiation exposure can be ensured.

The duration of neutron irradiation was prospectively determined at the start of irradiation based on real-time blood boron concentrations using a PK prediction model. Following the completion of irradiation, additional blood samples were collected to confirm the blood boron concentration during neutron irradiation and determine the delivered dose. These values were used to calculate the final delivered dose using the same PK model.

Blood samples were collected depending on the schedule and analyzed with inductively coupled plasma-mass spectrometry: before administration; 1 and 2 hours after the initiation of administration; 0, 10, 20, 30, 40, 50, and 60 minutes, as well as 2, 9, 24, and 48 hours after the completion of infusion. The PK in each patient was analyzed using a noncompartmental method with WinNonlin software 8.0 (Version 8.0, Certara). All the analyses were based on actual sampling times.

The neutron dose delivered to each patient was assessed as the biological dose (Gy-Eq), which was used as the primary reference metric for prescription and evaluation. Four physical dose components—boron, hydrogen, nitrogen, and gamma—were weighted by their respective relative biological effectiveness and compound biological effectiveness values and summed to yield the final biological dose.

For efficacy, the minimum tumoricidal dose was defined as ≥20 Gy-Eq to tumor tissue in a single fraction, based on linear–quadratic modeling. Therefore, eligibility required that ≥90% of the planning target volume be covered by at least 20 Gy-Eq, assuming a tumor-to-blood boron concentration ratio of ∼3.5.

For safety, dose escalation in this phase 1 trial was guided by the maximum absorbed dose to normal brain (D_max_), set at 9, 11, and 13 Gy-Eq across sequential cohorts. In other words, while tumor coverage of ≥20 Gy-Eq was mandated as an inclusion criterion, the prescribed dose levels (9-13 Gy-Eq) specifically referred to the maximum allowable dose to normal brain tissue, which served as the constraint for dose escalation.

The efficacy and safety were then evaluated for 6 months post-BNCT follow-up period, and the survival survey was conducted up to 2 years after BNCT, until the death of all patients, or the completion of the clinical trial, whichever came first.

### Statistical analysis

The primary endpoint was to determine RP2D by confirming MTD. Secondary endpoints were established to determine the safety, efficacy, and PK characteristics of BNCT.

The safety was evaluated by the number of patients, the incidence rate, and the number of cases for the AEs that occurred after the application of the investigational drug and device. The maximal severity analysis presented the number of patients who experienced treatment-emergent adverse events (TEAEs) and the incidence rate, coded according to system organ class and preferred term of MedDRA version 24.1 or a later version.

The efficacy evaluation included tumor response, such as progression-free survival (PFS), objective response rate, and OS, assessed by central imaging and the investigator using response assessment in neuro-oncology (RANO) and modified RANO (mRANO) criteria.

The blood concentration-time trend of ^10^B-BPA and ^10^B was plotted in linear or log/linear scale using R (version 4.3.3.) in each patient. The main PK parameters were calculated by noncompartmental method using the appropriate and validated PK software Phoenix WinNonlin (version 8.0, Certara). The PK parameters analyzed were as follows: C_max_, T_max_, AUC_last_ (area under the concentration–time curve, AUC, to the last measurable time point), AUC_0-∞_ (AUC extrapolated to infinity), CL (clearance), CL, _WT_ (weight-normalized clearance), t_1/2_, _z_, t_1/2, eff_, V_Z_, V_Z, WT_, V_SS_, V_SS, WT_, MRT (Mean residence time) of ^10^B-BPA and ^10^B, and CLR (renal clearance), A_e_ (amount unchanged excreted in urine), F_e_ (fraction excreted unchanged in urine) of ^10^B-BPA.

## Results

### Patient characteristics

**Figure 1:**
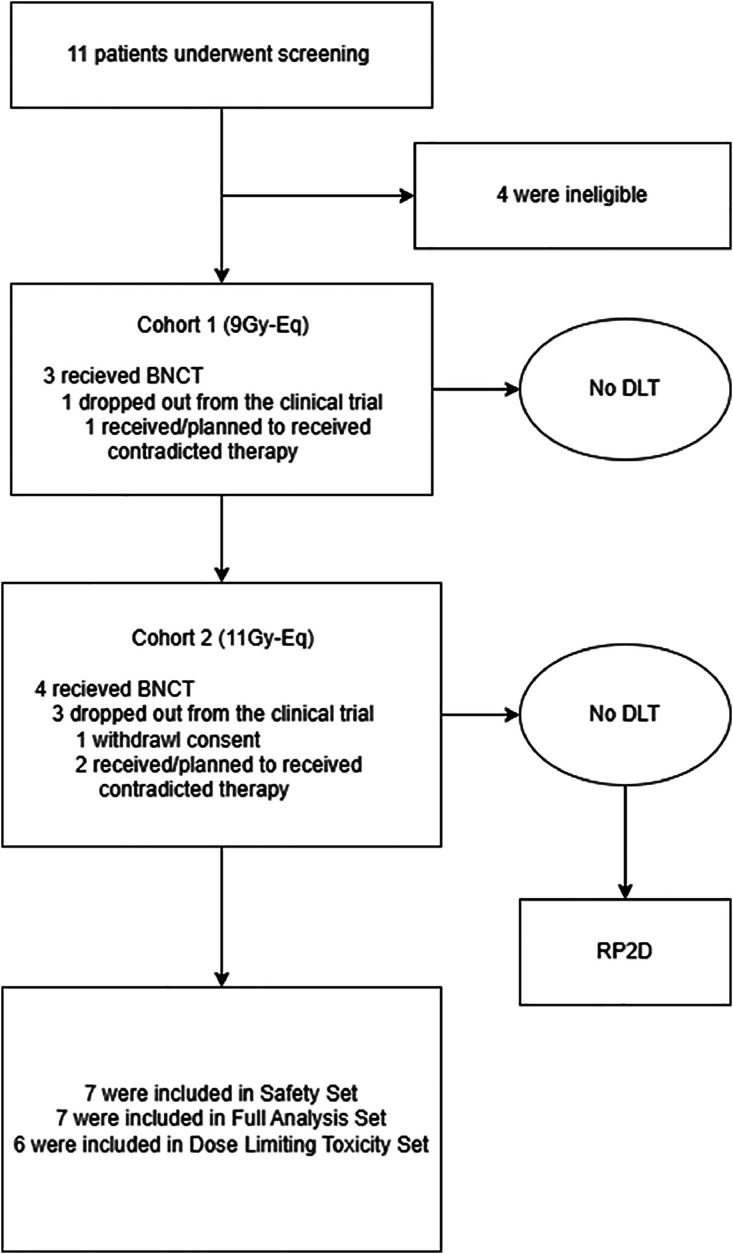
Flowchart of patient enrollment and analysis. *Abbreviations*: BNCT = boron neutron capture therapy; DLT = dose-limiting toxicity; RP2D = recommended phase 2 radiation dose.

Between December 2022 and January 2024, a total of 11 patients were screened after providing informed consent, and 7 of these patients were enrolled in this study and received BNCT. A total of 3 centers (Gil Medical Center, Incheon, South Korea; Seoul St. Mary's Hospital, Seoul, South Korea; National Cancer Center, Goyang, South Korea) were initially opened for screening, and 2 centers ultimately enrolled patients. All BNCT treatments were performed at the BNCT Center, Brain Research Center, Incheon, South Korea, where the BNCT facility is located. However, 1 patient from cohort 2 withdrew consent during neutron irradiation, having already shown a rapid cognitive decline immediately before the procedure, presumed to reflect aggressive progression of the recurrent lesion; this event was not considered BNCT-related, and the patient was therefore excluded from efficacy and safety analyses (Fig. 1, Table 1). The median age was 53 years (range, 39-57). The baseline characteristics and prior treatments of the included patients are listed in Table 1.

**Table 1:** Patient characteristics –full analysis set

Characteristics (units)	Cohort 1 (N = 3)	Cohort 2 (N = 3*)	All patients (N = 6)
Age, y			
Mean (SD)	47.0 (9.17)	54 (1.73)	50.9 (6.49)
Median (range)	45.0 (39-57)	55.0 (52-55)	53.0 (39-57)
Age group, n (%)			
<50	2 (66.67)	0	2 (33.33)
≥50	1 (33.33)	3 (100.00)	4 (66.67)
GTV (cc)			
Median (range)	9.3 (8.7-44.9)	32.1 (5.9-47.8)	20.7 (5.9-47.8)
Sex, n (%)			
Male	2 (66.67)	2 (66.67)	4 (66.67)
Female	1 (33.33)	1 (33.33)	2 (33.33)
KPS			
Median (range)	100.0 (90-100)	100.0 (90-100)	100.0 (90-100)
Diagnosis, n (%)			
Astrocytoma	0	1 (33.33)	1 (16.67)
Glioblastoma	3 (100.00)	2 (66.67)	5 (83.33)
WHO grade, n (%)			
4	3 (100.00)	3 (100.00)	6 (100.00)
RTOG RPA classification, n (%)			
2	1 (33.33)	0	1 (16.67)
3	2 (66.67)	0	2 (33.33)
4	0	1 (33.33)	1 (16.67)
5	0	2 (66.67)	2 (33.33)
Molecular genetic information, n (%)			
IDH-mutant	0	1 (33.33)	1 (16.67)
IDH wildtype	3 (100.00)	2 (66.67)	5 (83.33)
IDH-mutant, and 1p/19q-codeleted	0	0	0
Treatment history of primary disease, n (%)			
Radiation therapy	3 (100.0)	3 (100.0)	6 (100.0)
Chemotherapy	3 (100.0)	3 (100.0)	6 (100.0)
1st line	1 (33.33)	2 (66.67)	3 (50.00)
2nd line	2 (66.67)	0	2 (33.33)
3rd line or above	0	0	0
Missing	0	1 (33.33)	1 (16.67)
Immunotherapy	0 (0.0)	0 (0.0)	0 (0.0)
Salvage treatment	0 (0.0)	1† (33.33)	1 (16.67)
EBRT–BNCT interval (mo)‡			
Mean (range)	46.0 (27.9-66.6)	27.0 (5.5-46.9)	36.5 (5.5-66.6)

*Abbreviations:* BNCT = boron neutron capture therapy; EBRT = external beam radiation therapy; IDH = isocitrate dehydrogenase; KPS = Karnofsky performance status; RPA = recursive partitioning analysis; RTOG = Radiation Therapy Oncology Group; WHO = World Health Organization.

⁎ One patient from cohort 2 who withdrew consent on the day of neutron irradiation was excluded.

† Bevacizumab.

‡ Denotes interval from last EBRT fraction to BNCT start.

The median gross tumor volume was 20.7 cc with a range of 5.9 to 47.8 cc. The median Karnofsky performance status was 100 (range: 90-100). Five patients received diagnosis for GBM, and 1 patient had astrocytoma, World Health Organization (WHO) grade 4. Molecular genetic information showed that only 1 patient in cohort 2 was isocitrate dehydrogenase (IDH)-mutant, and all other patients were IDH wildtype, and WHO grade was grade 4 in all 6 patients. Radiation Therapy Oncology Group recursive partitioning analysis classification showed that classes 5 and 3 were the most prevalent at 33.33% (2/6), followed by classes 2 and 4 at 16.67% (1/6) each. On central imaging review, all patients demonstrated multiple nodular recurrences at baseline; however, no cases of leptomeningeal or extensive out-of-field dissemination were observed.

All patients had undergone prior surgical resection, radiation therapy, and chemotherapy. Before enrollment, second-line chemotherapy with temozolomide was administered to 2 patients (33.33%). One patient had previously received bevacizumab as part of salvage therapy. The interval between prior external beam radiation therapy and BNCT was a mean of 36.5 months (range, 5.5-66.6) across the enrolled population. The retrospectively determined delivered doses of normal and tumor tissues after BNCT, considering blood boron concentration and neutron beam parameter, are summarized in Table E1.

### Recommended phase 2 dose

No DLT occurred in cohorts 1 and 2. This outcome was achieved when the radiation dose could be escalated to 13 Gy-Eq, but the SMC decided 11 Gy-Eq as the RP2D without further dose escalation, based on the comprehensive safety and efficacy of each cohort.

### Safety outcomes and AEs

Following BNCT, a total of 1 case of serious adverse event (SAE), seizure, and 40 cases of TEAEs were observed in all 6 patients. TEAEs were categorized by maximum severity, using National Cancer Institute Common Terminology Criteria for Adverse Events version 5.0. Grade 2 TEAEs occurred in 4 patients (66.67%), and grade 3 TEAEs occurred in 2 patients (33.33%) (brain edema and seizure). No grade 4 or higher TEAEs nor treatment-related deaths were reported during the trial.

The most frequently reported TEAEs were alopecia, with 5 cases reported from 5 patients, and aphasia, with 5 cases in 3 patients. A total of 3 cases of paraesthesia were observed in 2 patients. In addition, 2 cases each of brain edema, seizure, hematuria, and parotitis, respectively, were reported from different patients (Table 2).

**Table 2:** Safety outcomes–safety set

	Cohort 1 (N = 3)		Cohort 2 (N = 3^+^)		All patients (N = 6)	
	N (%)		N (%)		N (%)	
Occurrence by maximum severity						
Grade 2	2 (66.67)		2 (66.67)		4 (66.67)	
Grade 3	1 (33.33)		1 (33.33)		2 (33.33)	

	N (%)	Cases	N (%)	Cases	N (%)	Cases

Patients experienced TEAEs	3 (100.00)	22	3 (100.00)	18	6 (100.00)	40
Alopecia	3 (100.00)	3	2 (66.67)	2	5 (83.33)	5
Aphasia	2 (66.67)	4	1 (33.33)	1	3 (50.00)	5
Paraesthesia	2 (66.67)	3	0	0	2 (33.33)	3
Brain edema	1 (33.33)	1	1 (33.33)	1	2 (33.33)	2
Parotitis	2 (66.67)	2	0	0	2 (33.33)	2
Seizure	0	0	2 (66.67)	2	2 (33.33)	2
Hematuria	1 (33.33)	1	1 (33.33)	1	2 (33.33)	2

*Abbreviation:* TEAE = treatment-emergent adverse event.

Consecutive occurrences of the same adverse event in 1 patient are considered 1 event.

MedDRA version 27.0.

### Efficacy outcomes

**Figure 2:**
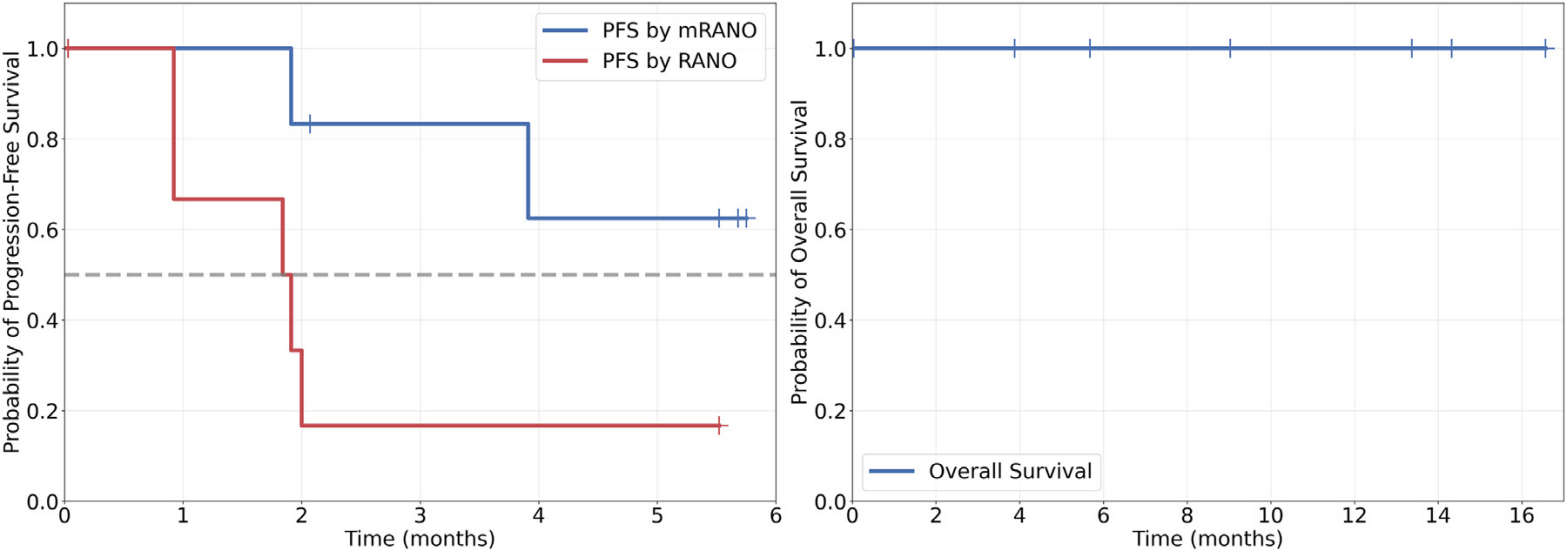
Progression-free survival (PFS) and overall survival outcomes–full analysis set. *Abbreviations*: mRANO = modified response assessment in neuro-oncology; RANO = response assessment in neuro-oncology.

No objective response was observed by either central imaging (RANO and mRANO) or investigator (mRANO) (Table 3). PFS was evaluated by central imaging and investigators using mRANO criteria; however, due to insufficient progression events per mRANO criteria, the median PFS was not reached (range, 1.91-5.75 months). The median PFS by central imaging with RANO criteria was 1.87 months (range, 0.92-5.52 months). The median follow-up period was 11.2 months. No deaths occurred during the follow-up period; therefore, median OS could not be ascertained (range, 3.88-16.56 months) (Fig. 2).

**Table 3:** ORR outcomes evaluated by central imaging according to modified RANO criteria –full analysis set

	Cohort 1 (N = 3)	Cohort 2 (N = 3^+^)	All patients (N = 6)
Best overall response (BOR), n (%)			
Confirmed CR	0	0	0
Confirmed PR	0	0	0
Confirmed PD	0	0	0
Stable disease (SD)	3 (100.00)	1 (33.33)	4 (66.67)
Not evaluable (NE)	0	2 (66.67)	2 (33.33)
Objective response rate (ORR)			
Confirmed CR or Confirmed PR, n (%)	0	0	0

*Abbreviations:* CR = complete response; PD = progression disease; PR = partial response; RANO = response assessment in neuro-oncology.

### PK outcomes

The ^10^B-BPA whole blood and ^10^B plasma concentrations measured during and after 3-hour intravenous infusion of DMX-101 at 500 mg/kg exhibited linear PK as shown in Fig. E2. The mean and SD (and range) of the CL of ^10^B-BPA in whole blood was 5.63 (±1.95) L/h, with a t_1/2, z_ of 8.65 (±3.39) h. The main PK parameters of whole blood ^10^B-BPA, plasma ^10^B, and urine ^10^B-BPA are presented in Table E2. The majority of ^10^B-BPA was excreted in the urine, with a mean value of F_e_ of 0.91, and almost all of it was excreted within 27 hours of dosing.

## Discussion

The treatment strategy for recurrent GBM or high-grade glioma (WHO grade 4 astrocytoma, IDH-mutant) varies based on recurrence patterns (diffuse, multifocal, or local), resectability, and patient performance status. While systemic therapies, reoperation, and reradiation therapy are available, clinical trials are often preferred due to unsatisfactory outcomes with current standard care.^8^ It is imperative to acknowledge the necessity for urgency and introduction of new technology, particularly in light of the nature of the high-grade glioma.

This study aimed to determine the RP2D for BNCT in previously irradiated recurrent high-grade glioma by escalating the brain dose (D_max_) from 9 Gy-Eq to 13 Gy-Eq. The selected dose range was based on previous studies. Coderre et al,^9^ after reviewing both the Massachusetts Institute of Technology (MIT) phase 1 study and the phase 1/2 study conducted at Brookhaven National Laboratory, concluded that the tolerance dose for the brain is approximately 14.1 Gy(W). This conclusion was derived from observations regarding the incidence of somnolence syndrome (D50/5), which was identified as the DLT. Furthermore, the P-03 study conducted in Finland established an upper limit by restricting brain doses to a maximum of 8 Gy(W).^10^ Additionally, a recent phase 2 trial conducted in Japan reported acceptable safety outcomes at a median administered dose of 10.9 Gy-Eq among previously irradiated patients. Moreover, equivalent dose in 2 Gy fractions calculations, commonly used^11^ to compare various radiation therapy dosing schedules by converting them into biologically equivalent doses, shows that doses of 9 to 13 Gy-Eq correspond to cumulative radiation doses approaching approximately 100 Gy.^12^^,^^13^

In this study, no DLTs were observed within the 90-day protocol-defined window up to 11 Gy-Eq. Under strict 3 + 3 rules, escalation to 13 Gy-Eq could technically have been pursued. However, the SMC considered additional safety signals that emerged beyond the formal DLT period. Specifically, in cohort 2, a grade 3 seizure occurred on day 98, and in cohort 1, 2 patients developed new 1 to 2 cm contrast-enhancing lesions with surrounding edema at approximately 8 and 10 months post-BNCT. These lesions showed no increased signal on perfusion magnetic resonance imaging and no specific uptake on ^18^F-FDOPA(^18^F‑fluoro‑L‑DOPA) PET/CT, supporting the clinical impression of radiation necrosis rather than progressive disease (PD). Both cases required bevacizumab and subsequently improved. Although these events were outside the central imaging review and formal DLT assessment, the SMC regarded them as clinically meaningful late toxicities.

Based on these qualitative but important observations, the committee judged that dose escalation might only accelerate the onset of radiation necrosis, thereby increasing long-term risk without offering incremental therapeutic gain. When integrating these findings with prior reirradiation BNCT constraints used in Finland^14^—which limited the normal brain peak dose to 8 Gy(W) and average dose to <6 Gy(W) while prescribing ≥17 Gy(W) to tumor—and with the Japanese^11^ multicenter phase 2 data reporting a median maximum dose to normal brain of 10.9 Gy-Eq (range, 9.6-11.6) alongside bevacizumab-controlled edema after AB-BNCT in recurrent GBM, the SMC considered escalation beyond 11 Gy-Eq to confer undue risk without clear incremental benefit. Accordingly, 11 Gy-Eq was conservatively recommended as the RP2D.

In consistency with previous BNCT trials,^11^ the most common TEAEs were alopecia, aphasia, brain edema, and seizures, caused by pseudoprogression or radiation-induced necrosis. Notably, episodes of aphasia were observed predominantly in patients with tumors adjacent to the eloquent cortex or with peritumoral edema, suggesting that these were attributable to local tumor- and edema-related effects rather than nonspecific systemic toxicity of BNCT. Appropriate countermeasures with bevacizumab and corticosteroids against these predictable radiation-induced pseudoprogression or necrosis will make the symptom tolerable; however, the limitations of long-term corticosteroid use underscore the potential value of BNCT–bevacizumab synergy. Indeed, Miyatake et al^1^ reported that bevacizumab following BNCT prolonged mPFS and OS, supporting a potential therapeutic benefit when combined with anti-vascular endothelial growth factor therapy.^15^^,^^16^

Although bevacizumab was prohibited in the present study to distinctly assess BNCT’s safety profile, these observations highlight why symptomatic pseudoprogression became a central clinical concern, directly impacting the Safety Committee’s RP2D recommendation. A phase 2 trial that permits on-demand bevacizumab after BNCT is now ongoing to clarify this combination’s role.

Another salient consideration is the discrepancy between RANO and mRANO criteria, which may lead to misclassification of pseudoprogression as true progression. This is particularly relevant for BNCT, where transient lesion enlargement after treatment can mimic progression and complicate clinical decision-making. Such challenges, however, can be mitigated by specialized expertise and the application of modified assessment criteria, underscoring the importance of careful interpretation in both safety evaluation and trial design.

In the current BNCT trial, the observed objective response rate was 0%. The median PFS was 1.87 months by RANO criteria and not reached by mRANO criteria. These clinically contradictory results are primarily attributable to symptomatic pseudoprogression—findings that are commonly observed following radiation therapy, underscoring the necessity of differentiating between genuine therapeutic response and pseudoprogression.

**Figure 3:**
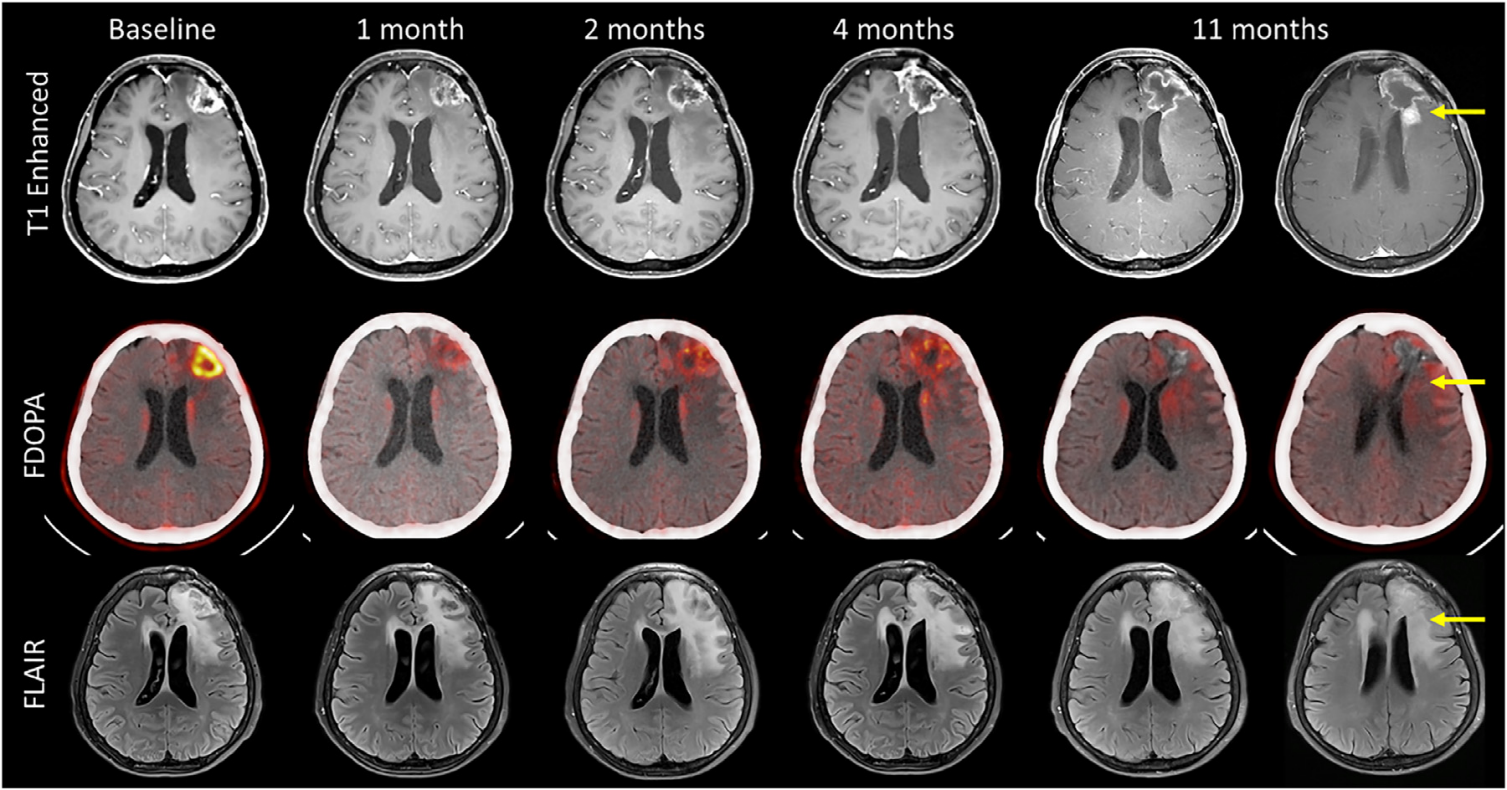
Key example. A 57-year-old female was diagnosed as glioblastoma multiforme with IDH wildtype grade 4, RPA II, MGMT methylated, and TERT-mutant. The patient received tumor resection, radiation therapy, and chemotherapy with temozolomide. Four months after BNCT, a low dose of bevacizumab was administered to control the symptoms of cerebral edema that had increased 2 to 4 months after the BNCT, which accompanies tumor necrosis due to the large tumor size. This case demonstrates the tumor control capability of BNCT, as the patient, despite being a rapidly progressing case with a new 2 to 3 cm tumor detected at the resection margin just 4 months after surgery, remained in a stable condition without evidence of recurrence until 11 months posttreatment. *Abbreviations*: BNCT = boron neutron capture therapy; FDOPA = 6-[18F]fluoro-L-3,4-dihydroxyphenylalanine; FLAIR = fluid-attenuated inversion recovery; IDH = isocitrate dehydrogenase; MGMT = O6-methylguanine-DNA methyltransferase; RPA = recursive partitioning analysis; TERT = telomerase reverse transcriptase.

For example, in patients presenting with a significant tumor burden, a considerable tumor reduction leading to cavitation, and the emergence of marginal linear enhancement along the cavity wall—where the tumor mass was previously located—with minimal residual FDOPA uptake observed post-BNCT. Notwithstanding this finding, the cases were classified as PD by independent reviewers at 1 month per RANO and at 4 months per mRANO post-BNCT due to lesion enlargement (Fig. 3). This observation illustrates a potential discordance between radiographic interpretation and underlying biological response. Such radiological response patterns were observed throughout the present study, emphasizing the critical need to accurately distinguish true tumor progression from pseudoprogression.

Likewise, Kawabata et al^11^ reported the results of a phase 2 trial of accelerator-based BNCT in patients with previously irradiated recurrent GBM. The median PFS by RANO was only 0.9 months, while the median OS (mOS) was 18.9 months.^11^ This marked disparity between radiological progression and clinical outcome supports the frequent occurrence of pseudoprogression following BNCT and further illustrates the challenge in accurately evaluating true biological response.

In the current study, mRANO was employed to assess whether early progression observed per RANO could, in fact, be indicative of pseudoprogression, as mRANO criteria allow for extended observation before confirming PD.^17^ In certain cases, such as the one illustrated in Fig. 3, mRANO enabled a longer PFS determination of up to 4 months, though radiologic interpretation tended to classify the lesion as PD due to cavitation and apparent lesion enlargement. Recognizing these limitations, RANO 2.0—which incorporates decision logic similar to mRANO—will be adopted in the upcoming trial to better differentiate true tumor progression from pseudoprogression in the context of BNCT.^18^

As all patients remained alive at the time of data cutoff, the median OS was not reached, underscoring the limitations of interpretation given the short follow-up period. In light of the fact that the current phase 1 trial primarily focused on short-term safety evaluation, the median follow-up period was 11.2 months, and no mortalities were reported during this period. The interpretation of these results must be conducted with a high degree of caution. This is due to the fact that the follow-up duration and the post-trial observation period are limited, which has the potential to introduce bias when estimating true long-term survival.

However, it is important to note that there are several limitations associated with this approach. The study's limitations, including its modest sample size and single-arm design, restrict the extent to which its findings can be generalized. Moreover, the absence of a comparator arm precludes definitive conclusions about BNCT's superiority over existing treatments. Another limitation is the short follow-up period, which restricts the assessment of long-term survival and late toxicities. Additionally, a single-arm phase 2 trial (KCT0010193) is currently underway to evaluate in recurrent GBM. This trial plans to recruit a larger patient cohort and extend the follow-up period, allowing for a more robust evaluation of the treatment's effectiveness. Furthermore, the potential of imaging biomarkers (^18^F-FDOPA PET/CT or ^18^F-FET PET/CT) will be investigated to assess their clinical usefulness in identifying patients most likely to benefit from BNCT. This exploration has the potential to optimize patient selection and improve outcomes in future studies.

## Conclusion

BNCT demonstrates potential as a novel therapeutic approach for recurrent high-grade gliomas. No DLT was observed across both cohorts, along with the consistency of TEAEs with those previously reported in BNCT studies, which substantiates the safety profile. This phase 1 trial establishes a preliminary foundation for subsequent research, emphasizing the need to further explore its long-term safety and efficacy in larger, more diverse populations.

## Disclosures

Gi-Taek Yee reports financial support was provided by Dawonmedax Co Ltd Heon Yoo, Hyung-Seok Lim, Hyun Ju Kim, Jae-Sung Park, Jin-Ho Song, JunGyu Yi, Kawngwoo Park, Seunggyun Ha, Stephen Ahn, Sung Uk Lee, and Dong-Won Shin report financial support was provided by Dawonmedax Co Ltd Gi-Taek Yee reports a relationship with Dawonmedax Co Ltd that includes: consulting or advisory. Heon Yoo and Hyung-Seok Lim report a relationship with Dawonmedax Co Ltd that includes: consulting or advisory. Kwan Cho, JunGyu Yi, Hyo Jung Seo, and Woohyoung Kim report a relationship with Dawonmedax Co Ltd that includes: employment. Dong-Won Shin served as subinvestigator in a sponsor-initiated trial funded by Dawonmedax Co, Ltd; no employment, consulting fees, stock, or other industry relationships declared. Gi-Taek Yee principal investigator in trials sponsored by Dawonmedax Co, Ltd and Rzinomix; no additional industry employment or consulting relationships reported. Heon Yoo principal investigator in trials sponsored by Dawonmedax Co, Ltd, PPD Development Korea, and CellabMed Ltd; declares no consulting fees, stock, or other industry interests. Hyeong-Seok Lim received research funding from Dawonmedax Co, Ltd for pharmacokinetic model development; no investigator roles or consulting activities disclosed. Hyo Jung Seo full-time employee and shareholder of Dawonmedax Co, Ltd; participated in company-sponsored research and holds leadership roles in several professional societies; no external consulting fees declared. Jae-Sung Park principal investigator in sponsor-initiated trials funded by Dawonmedax Co, Ltd, Chimerix Inc, and Polaris Pharmaceuticals Inc; no consulting or stock ownership declared. Jin-Ho Song subinvestigator in a Dawonmedax-sponsored trial; reports no other financial or advisory relationships with industry. JunGyu Yi full-time employee and shareholder of Dawonmedax Co, Ltd; sponsor provided funding, study materials, and internal medical-writing support for this manuscript; no external consulting. Kwan Cho full-time employee of Dawonmedax Co, Ltd; member of the study Safety Monitoring Committee and medical-advisory board; no additional industry funding or royalties. Woo-Hyung Kim full-time employee of Dawonmedax Co, Ltd; member of the study Safety Monitoring Committee and medical-advisory board; previously employed by Samsung Bioepis; served as (sub-/principal) investigator in Dawonmedax-sponsored trials; currently no paid consulting engagements disclosed. The other authors declare that they have no known competing financial interests or personal relationships that could have appeared to influence the work reported in this paper.

## Appendix Supplementary materials

Supplementary Data_Revised_1009 Image, application 1

## Footnotes

Supplementary material associated with this article can be found in the online version at doi:10.1016/j.adro.2025.101947.
