# Supplementary material for: Boron Neutron Capture Therapy in Recurrent High-Grade Gliomas: Safety, Efficacy, and Pharmacokinetics From a Multicenter, Dose-Escalation Phase 1 Trial
Source: Adv Radiat Oncol. 2025 Oct 30;11(1):101947. doi: 10.1016/j.adro.2025.101947 (PMC12719081; doi:10.1016/j.adro.2025.101947)
Supplement: Supplementary Data_Revised_1009 [file mmc1.docx]

**Supplementary Figures and Legends**


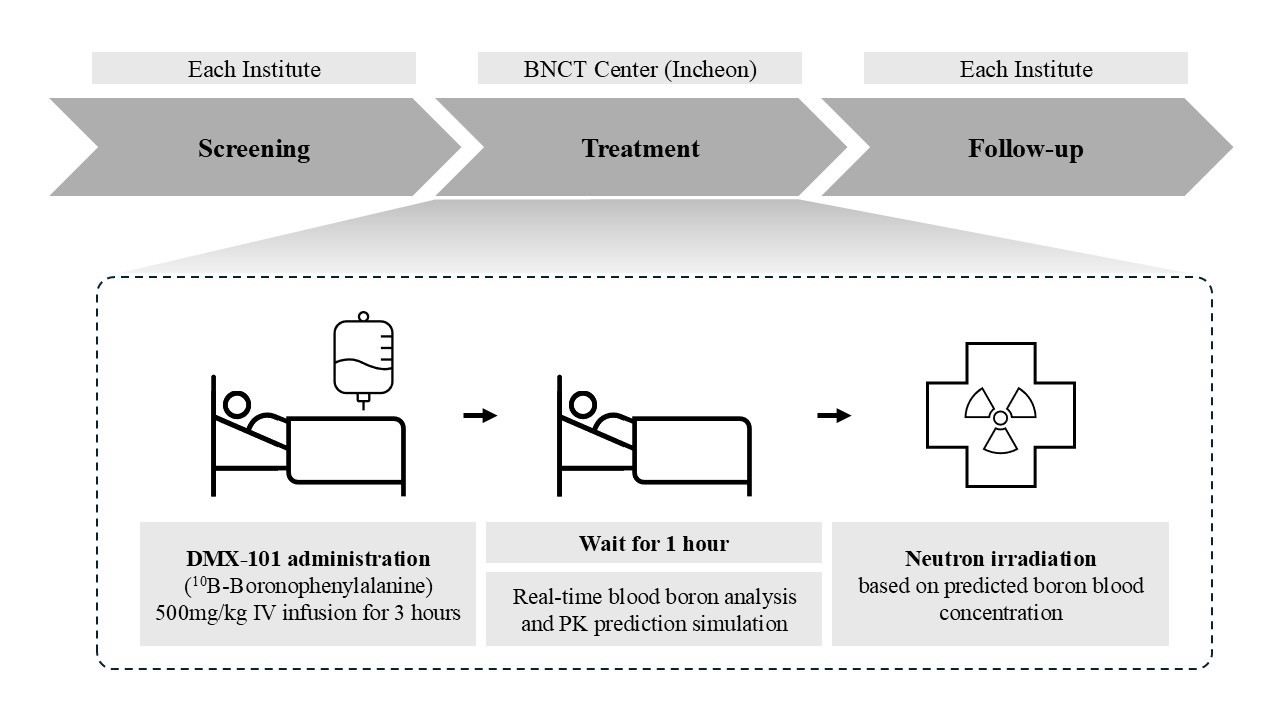


**Supplementary Figure E1.** Protocol Overview


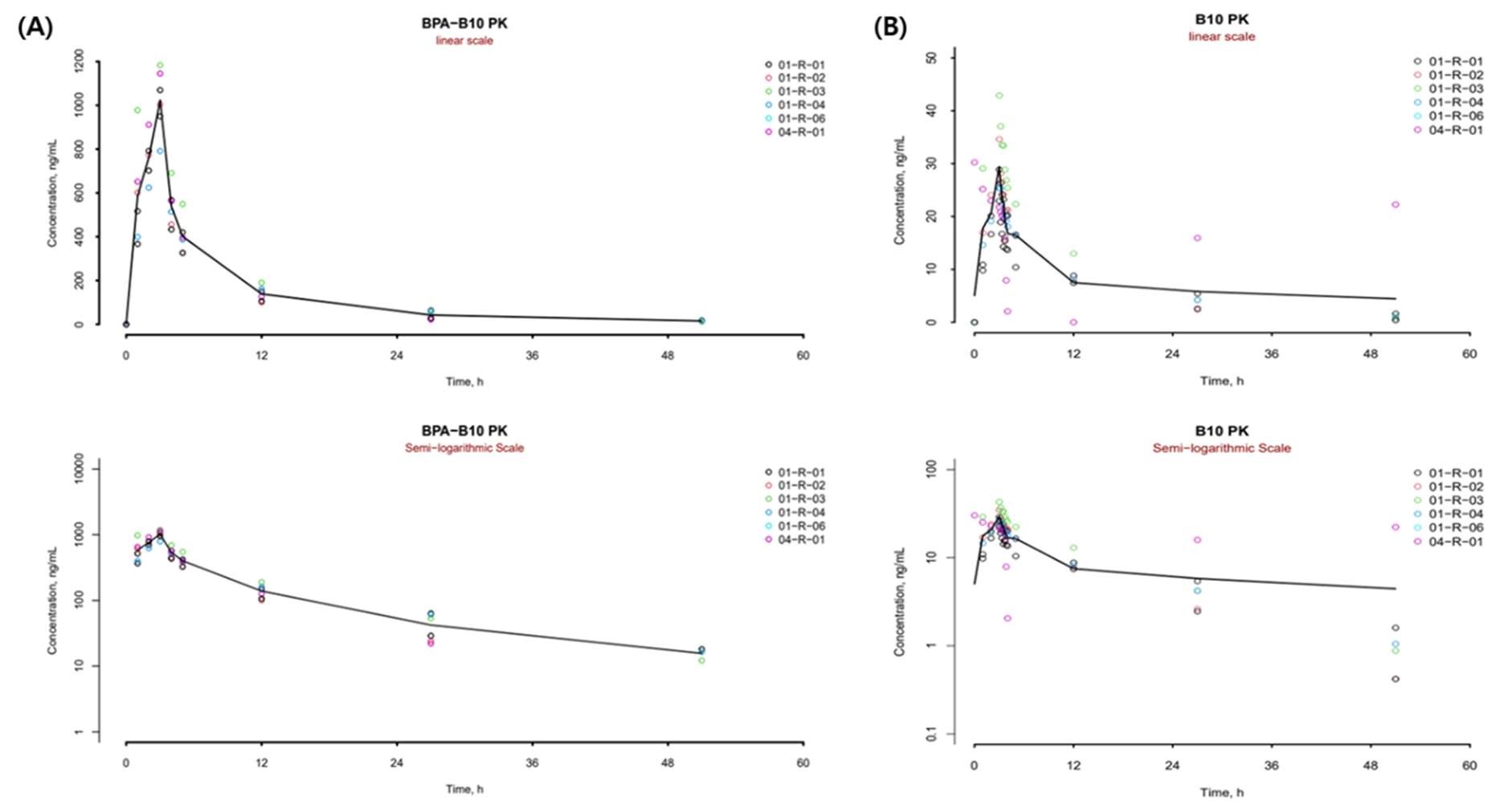


**Supplementary Figure E2.** Mean Plasma Concentration Time Curves of ^10^B-BPA(A) and ^10^B (B)

**Supplementary Table LEGENDs**

**Supplementary Table E1.** Dose of Normal Organs and Tumor based on B-10 Concentration after Neutron Irradiation

| **Contours** | **Cohort 1 (n=3) Median (Range)** | **Cohort 2 (n=3) Median (Range)** | **Total (n=6) Median (Range)** |  |
| --- | --- | --- | --- | --- |
| Gross Tumor Volume |  |  |  |  |
| Minimum | 3328.1  (3104.2-4536.0) | 3174.3  (2147.6-3243.0) | 3208.7  (2147.6-4536.0) |  |
| Maximum | 5602.4  (5399.0-6341.4) | 5232.4  (5197.6-5854.7) | 5500.7  (5197.6-6341.4) |  |
| Median | 5286.4  (4611.8-5383.0) | 4152.0  (3944.8-4413.7) | 4512.8  (3944.8-5383.0) |  |
| Major Organs or Tissue |  | | |  |
| Brain (D_max_) | 878.8  (878.8-894.7) | 1104.5  (1083.7-1156.4) | 989.2  (878.8-1156.4) |  |
| Brain (Mean) | 248.4  (240.1-259.3) | 311.8  (309.5-335.3) | 284.4  (240.1-335.3) |  |
| Brain stem (D_max_) | 230.9  (183.5-371.0) | 343.8  (286.2-503.0) | 315.0  (183.5-503.0) |  |
| Optic chiasm (D_max_) | 259.0  (153.7-262.1) | 273.8  (136.2-443.4) | 260.6  (136.2-443.4) |  |
| Optic Nerve (D_max_) | 308.0  (137.0-344.0) | 352.1  (117.7-446.9) | 326.0  (117.7-446.9) |  |
| Eyeball (D_max_) | 378.5  (134.7-387.9) | 400.0  (75.6-705.7) | 383.2  (75.6-705.7) |  |
| Lens (D_max_) | 233.7  (75.9-267.0) | 226.9  (37.1-255.4) | 230.3  (37.1-267.0) |  |
| Mucosa (oral cavity, larynx and pharynx) (D_max_) | 112.2  (44.8-149.4) | 200.9  (51.8-407.7) | 130.8  (44.8-407.7) |  |
| Analyzed for all patients who completed neutron irradiation (excluding patient who withdrew consent) | | | | |

All values are reported as BNCT-equivalent dose in cGy-Eq (1 Gy-Eq = 100 cGy-Eq).

**Supplementary Table E2.** Noncompartmental PK analysis result of blood ^10^B BPA, plasma ^10^B and urine ^10^B-BPA

| Median  (Min-Max) | C_max_  (mg/L) | T_max_  (h) | AUC_last_  (ng*h/mL) | AUC_0-∞_  (h*mg/mL) | t_1/2,z_  (h) | t_1/2,eff_  (h) | CL  (L/h) | Vz  (L) | CL_R_  (L/h) |
| --- | --- | --- | --- | --- | --- | --- | --- | --- | --- |
| ^10^B-BPA | 1037.0  (791.5-1183.6) | 3.1  (3.0-3.2) | 6321.7  (5074.0-8767.0) | 6543.2  (5302.6-8933.1) | 8.0  (5.4-12.9) | 5.3 (3.8-8.8) | 5.9  (3.1-7.7) | 62.3  (42.6-118.4) | 5.4  (2.2-9.9) |
| ^10^B | 29.6  (22.9-42.9) | 3.1  (3.0-3.2) | 296.1  (224.2-428.9) | 309.1  (229.9-441.0) | 9.7  (7.4-14.5) | 8.7 (6.61-13.54) | 126.7  (62.8-173.8) | 1675.1  (895.2-2575.0) | - |

C_max_, maximal concentration; T_max_, time to peak concentration; AUC_last_, area under the curve to the last measurable concentration; AUC_0-∞_, Area under the curve to infinity; t_1/2,eff_, effective half-life; CL, clearance; V_Z_, volume of distribution during terminal phase; CL_R_, renal clearance; F_e,WT_, weight normalized fraction excreted unchanged in urine

**Supplementary Information**

Definition of Dose-Limiting Toxicity (DLT)

DLTs in this trial were evaluated by the Safety Monitoring Committee (SMC) based on adverse events (AEs) occurring within 90 days after BNCT. The decision to escalate the dose (defined by the maximum absorbed dose to normal brain) or to confirm the MTD was made according to these findings.

A DLT was defined as any BNCT-related AE (as determined by causality assessment, per protocol Section 15.1.2) that occurred within 90 days post-treatment, according to the National Cancer Institute Common Terminology Criteria for Adverse Events (NCI CTCAE) version 5.0.

1. Non-hematologic toxicities

Any Grade ≥3 non-hematologic toxicity was considered a DLT. Specific definitions included:

• Cerebral edema with symptoms: Grade 3 cerebral edema was defined as:

1. severe or medically significant but not immediately life-threatening symptoms (e.g., headache, vomiting, altered level of consciousness, focal neurological deficits, visual disturbance, or other related neurological manifestations); or
2. cerebral edema requiring hospitalization or prolongation of an existing hospitalization; or
3. substantial limitation of self-care; or
4. inability to perform self-care activities of daily living (ADL), which include bathing, dressing and undressing, feeding oneself, using the toilet, and taking medications.

• Serum amylase elevation was excluded from DLT determination.

2. Hematologic toxicities

Any Grade ≥3 hematologic toxicity was considered a DLT. However, isolated laboratory abnormalities without clinical significance (e.g., lymphopenia) were reviewed by the SMC to determine whether they constituted a DLT.
